# Cerebrospinal fluid proteomic associations of *APOE* genotypes reveal distinct protective and risk mechanisms for Alzheimer's disease

**DOI:** 10.1002/alz.70738

**Published:** 2025-10-14

**Authors:** Eleonora M. Vromen, Senne B. Lageman, Johan Gobom, Rik van der Kant, Valerija Dobricic, Lars Bertram, Johannes Streffer, Simon Lovestone, Stephanie J. B. Vos, Mikel Tainta, Yvonne Freund‐Levi, Lutz Frölich, Julius Popp, Gwendoline Peyratout, Magda Tsolaki, Frans Verhey, Rik Vandenberghe, Jolien Schaeverbeke, Kaj Blennow, Sven J. van der Lee, Philip Scheltens, Yolande A. L. Pijnenburg, Wiesje M. van der Flier, Charlotte E. Teunissen, Henrik Zetterberg, Pieter Jelle Visser, Betty Tijms

**Affiliations:** ^1^ Alzheimer Center Amsterdam Neurology Vrije Universiteit Amsterdam Amsterdam UMC location VUmc Amsterdam the Netherlands; ^2^ Amsterdam Neuroscience Neurodegeneration Amsterdam the Netherlands; ^3^ Clinical Neurochemistry Laboratory Sahlgrenska University Hospital Mölndal Sweden; ^4^ Department of Psychiatry and Neurochemistry Institute of Neuroscience and Physiology Sahlgrenska Academy at the University of Gothenburg Mölndal Sweden; ^5^ Lübeck Interdisciplinary Platform for Genome Analytics (LIGA) University of Lübeck Lübeck Germany; ^6^ Department of Biomedical Sciences Campus Drie Eiken University of Antwerp Antwerp Belgium; ^7^ Department of Psychiatry University of Oxford Warneford Hospital Oxford UK; ^8^ Janssen Pharmaceutica Beerse Belgium; ^9^ Alzheimer Centrum Limburg Department of Psychiatry and Neuropsychology Maastricht University Maastricht the Netherlands; ^10^ Neurology Department CITA Alzheimer Foundation San Sebastian Spain; ^11^ Zumarraga Hospital OSI Goierri‐Alto Urola Osakidetza Zumarraga Spain; ^12^ Department of Clinical Science and Education Södersjukhuset Karolinska Institutet Stockholm Sweden; ^13^ Department of Geriatrics Department of Medical Science Örebro University University Hospital Örebro and Södertälje Hospital Örebro Sweden; ^14^ Department of Geriatric Psychiatry Central Institute of Mental Health Medical Faculty Mannheim Heidelberg University Mannheim Germany; ^15^ University Hospital of Psychiatry and University of Zurich Zürich Switzerland; ^16^ Service of Old Age Psychiatry Department of Psychiatry University Hospital of Lausanne Prilly Switzerland; ^17^ Third Department of Neurology Aristotle University of Thessaloniki Thessaloniki Greece; ^18^ Neurology Service University Hospitals Leuven Leuven Belgium; ^19^ Laboratory for Cognitive Neurology Department of Neurosciences KU Leuven Leuven Belgium; ^20^ Laboratory of Neuropathology Leuven Brain Institute KU Leuven Leuven Belgium; ^21^ Paris Brain Institute ICM Pitié‐Salpêtrière Hospital Sorbonne University Paris France; ^22^ Neurodegenerative Disorder Research Center Division of Life Sciences and Medicine and Department of Neurology Institute on Aging and Brain Disorders University of Science and Technology of China and First Affiliated Hospital of USTC Hefei P.R. China; ^23^ Section Genomics of Neurodegenerative Diseases and Aging Department of Clinical Genetics Vrije Universiteit Amsterdam Amsterdam UMC Amsterdam the Netherlands; ^24^ Department of Epidemiology and Data Sciences Vrije Universiteit Amsterdam Amsterdam UMC Amsterdam the Netherlands; ^25^ Neurochemistry Laboratory Department of Laboratory Medicine Amsterdam Neuroscience Vrije Universiteit Amsterdam Amsterdam UMC Amsterdam the Netherlands; ^26^ Department of Neurodegenerative Disease UCL Institute of Neurology London UK; ^27^ UK Dementia Research Institute, UCL London UK; ^28^ Hong Kong Center for Neurodegenerative Diseases Hong Kong Science Park Hong Kong P.R. China; ^29^ Wisconsin Alzheimer's Disease Research Center University of Wisconsin School of Medicine and Public Health University of Wisconsin–Madison Madison Wisconsin USA; ^30^ Department of Neurobiology Care Sciences and Society Division of Neurogeriatrics Karolinska Institutet BioClinicum Solna Sweden

**Keywords:** Alzheimer's disease, apolipoprotein E, cerebrospinal fluid, cognitively normal, mild cognitive impairment, protection, proteomics, risk

## Abstract

**BACKGROUND:**

The apolipoprotein E (*APOE*) gene includes the strongest protective (ε2) and risk (ε4) variants for sporadic Alzheimer's disease (AD), but underlying mechanisms remain unclear. We studied *APOE* genotype effects on the cerebrospinal fluid (CSF) proteome.

**METHODS:**

Using untargeted tandem mass tag mass spectrometry, we analyzed CSF from 227 cognitively normal (CN) controls (A–T–), 165 CN A+, and 177 individuals with mild cognitive impairment (MCI A+) from two large cohorts. We compared protein levels across *APOE* genotypes using linear regression and characterized biological pathways.

**RESULTS:**

Five hundred forty‐nine of 978 proteins (56%) differed between ε2/ε3 (*n* = 32 individuals) or ε4 carriers (*n* = 181 individuals) and ε3/ε3 controls. ε2/ε3 controls showed the most differences, with higher levels of 280 proteins enriched for neuronal plasticity. ε4 carrier controls showed increased proteins linked to blood–brain barrier dysfunction, and A+ ε4 carriers were related to glucose metabolism.

**DISCUSSION:**

Combining two cohorts enabled analysis of the rare *APOE* ε2 genotype, suggesting protective effects may occur through improved neuronal plasticity.

**Highlights:**

- Apolipoprotein E (*APOE*) genotypes show distinct cerebrospinal fluid proteomic mechanisms in early Alzheimer's disease (AD).
- Combining cohorts enabled analysis of rare *APOE* ε2–associated protection in AD.
- The rare ε2 genotype may confer protection through improved neuronal plasticity.
- *APOE* ε4 carriers show increased blood–brain barrier dysfunction and glucose metabolism.
- These findings offer new insights into genotype‐specific mechanisms in early AD.

## 1 BACKGROUND

The apolipoprotein E (*APOE*) gene is the major genetic risk and protective factor for Alzheimer's disease (AD).^1^, ^2^ This gene has three alleles: ε2, ε3, and ε4, of which, compared to homozygous ε3 individuals, the ε4 allele increases AD dementia risk ≈ 3‐fold for individuals carrying one ε4 allele to > 14‐fold for individuals carrying two ε4 alleles.^1^ Furthermore, ε4 decreases the age of disease onset.^1^, ^3^ In contrast, the ε2 allele is protective: ε2/ε3 carriers have a 1.5‐ to 3‐fold reduced risk, and ε2/ε2 carriers have a 2.5‐ to 7.5‐fold reduced risk compared to ε3/ε3 individuals.^1^, ^4^, ^5^ Note, however, that these protective genotypes are relatively rare, with ε2/ε3 occurring in roughly 10% to 15% and ε2/ε2 in only 0.2% to 1.4% of the general population.^4^, ^5^, ^6^, ^7^ *APOE* has been implicated in numerous pathways that contribute to AD pathology, including amyloid metabolism and clearance pathways,^8^ synaptic plasticity, lipid and glucose metabolism, and neuroinflammation.^9^ Yet, the precise molecular processes through which different *APOE* alleles affect AD pathophysiology in patients remain largely unknown, while such information may provide more insight into treatments that may help reduce risk for AD.

One approach to studying ongoing biological processes in the brain is through cerebrospinal fluid (CSF) proteomic analyses. One of the first CSF proteomic studies investigating *APOE* effects included 243 targeted proteins in 193 subjects who had abnormal amyloid and reported altered CSF levels of proteins associated with *APOE* ε4 that were involved in neuronal injury, cell adhesion, and inflammatory processes. These *APOE* ε4–related protein changes were dependent on clinical stage.^10^ Furthermore, another group reported, in part of the same data, decreases in immune‐related protein levels with ε4 dose, suggesting that higher ε4 dose aggravates involved processes, but this study did not take into account clinical stage.^11^ It remains unclear to what extent those results generalize to other cohorts and if additional processes may be detected when increasing the number of proteins studied. Furthermore, the mechanisms through which the *APOE* ε2 allele may contribute to protection against AD remain unclear, as research into this question is restricted by its low prevalence, especially in AD populations.^4^, ^12^ Most knowledge on ε2‐related mechanisms is based on animal or cell studies, the results of which suggest that protective mechanisms may be related to a more efficient amyloid clearance, altered lipid metabolism, and neurotrophic effects to maintain synaptic functioning.^5^, ^13^, ^14^, ^15^ New proteomic techniques now enable detection of thousands of proteins in the CSF, offering detailed studies into molecular mechanisms related to *APOE* genotypes. It could be hypothesized that these mechanisms of amyloid clearance, lipid metabolism, and synaptic functioning may be reflected in *APOE* genotype‐related alterations. While ε4 is enriched in AD populations, the low prevalence of ε2 requires very large sample sizes, on the other hand, to study potential protective effects, but such large‐scale CSF studies are rare.

**RESEARCH IN CONTEXT**

1. **Systematic review**: The authors reviewed relevant literature using traditional sources (e.g., PubMed) to examine how apolipoprotein E (*APOE*) genotype influences Alzheimer's disease (AD) pathophysiology through cerebrospinal fluid (CSF) proteomics. While ε4‐related risk mechanisms have been studied extensively, knowledge on ε2‐related protective pathways in patients remains limited due to its low population frequency.
2. **Interpretation**: By combining CSF proteomics data from two cohorts, we were able to create a large enough sample size to study biological pathways related to the rare *APOE* ε2 allele in patients. The results suggest that potential protective effects may be mediated through processes related to neuronal plasticity. These findings provide new insights into genotype‐specific mechanisms in early AD.
3. **Future directions**: Longitudinal studies with repeated CSF sampling are needed to examine how these proteomic profiles evolve over time and relate to amyloid and tau pathology to test whether these pathways contribute to protection or risk.

In this study, we took advantage of such approaches that were applied in two large‐scale cohorts: the clinical Amsterdam Dementia Cohort (ADC)^16^ and the multi‐center European Medical Information Framework‐AD Multimodal Biomarker Discovery study (EMIF‐AD MBD),^17^ which, by pooling these together, enabled us to create a sample size large enough to study the potential mechanisms through which ε2 may protect against disease. We further studied early AD alterations reflected in CSF in both cognitively normal (CN) and mild cognitively impaired (MCI) participants.

## 2 MATERIALS AND METHODS

### 2.1 Participants and cohorts

We selected participants from the clinical ADC^16^ and from the multi‐center EMIF‐AD MBD^17^ with CSF proteomic measurements available. In total, we included 165 CN A+ individuals (i.e., cognitively normal or subjective cognitive decline and at least abnormal CSF amyloid; *n* = 98 ADC, *n* = 67 EMIF‐AD MBD), 177 individuals with MCI A+ (i.e., MCI and at least abnormal CSF amyloid; *n *= 97 ADC, *n* = 80 EMIF‐AD MBD), and 227 CN individuals with normal CSF AD biomarkers (i.e., controls; *n* = 166 ADC, *n* = 61 EMIF‐AD MBD). Controls with *APOE* ε3/ε3 (*n* = 143) were used as reference group for the main analysis. Diagnosis of MCI was based on international criteria.^18^, ^19^, ^20^ These studies were approved by local medical ethical committees.

### 2.2 *APOE* analysis

*APOE* genotyping was performed in blood using the QIAxcel DNA Fast Analysis kit (Qiagen) in ADC,^21^ and in EMIF‐AD MBD it was assessed using genome‐wide single nucleotide polymorphism (SNP) genotyping^22^ based on the combination of the SNP determining the *APOE* ε4 allele (rs429358) and the SNP determining the *APOE* ε2 allele (rs7412). For our main analysis we excluded all individuals with *APOE* ε2/ε4 and MCI A+ individuals with *APOE* ε2/ε3 because of the small group sizes (*n* ≤ 6, but we report descriptive values in Table S1 in supporting information for completeness). For our secondary analysis, we coded *APOE* genotype as a continuous variable to reflect increasing degrees of genetic risk as follows: ε2/ε3 as –1, ε3/ε3 as 0, both ε2/ε4 and ε3/ε4 as 1—as these show similar genetic risk for AD^4^—and ε4/ε4 as 2.

### 2.3 CSF analysis

Lumbar puncture was performed to collect CSF in polypropylene tubes, preferably using a small‐gauge atraumatic needle, and processed according to international criteria.^23^, ^24^ In ADC, concentrations of amyloid beta (Aβ) 1‐42 (Aβ42), phosphorylated tau 181 (p‐tau), and total tau (t‐tau) were determined using enzyme‐linked immunosorbent assay (ELISA; INNOTEST β‐AMYLOID,^1^, ^2^, ^3^, ^4^, ^5^, ^6^, ^7^, ^8^, ^9^, ^10^, ^11^, ^12^, ^13^, ^14^, ^15^, ^16^, ^17^, ^18^, ^19^, ^20^, ^21^, ^22^, ^23^, ^24^, ^25^, ^26^, ^27^, ^28^, ^29^, ^30^, ^31^, ^32^, ^33^, ^34^, ^35^, ^36^, ^37^, ^38^, ^39^, ^40^, ^41^, ^42^ PHOSPHO‐TAU[181P], and HTAU‐AG; Fujirebio) as described previously.^25^ Amyloid abnormality was defined by using a drift‐corrected cutoff of < 813 pg/mL.^26^ In EMIF‐AD MBD, study centers used different methodologies to determine biomarker concentrations. Center‐specific cutoffs for amyloid were previously established using unbiased Gaussian mixture modeling.^27^

In both cohorts, proteomics was performed with tandem mass tag mass spectrometry (TMT‐MS), in ADC with 16‐plexing and in EMIF‐AD MDB with 10+1 plexing, both using a high pH reverse phase high‐performance liquid chromatography for peptide prefractionation. Reference channels were used to normalize peptide relative abundances between TMT experiments, according to standard procedures.^28^ Further details regarding CSF sample preparation and MS analysis have been described previously.^27^, ^29^ For both cohorts, protein concentrations were first log‐transformed and subsequently scaled according to the mean and standard deviation values of the control group (defined as A−T− individuals with *APOE* ε3/ε3 genotype) to aid comparisons between proteins and cohorts. We selected proteins for the present study when they were detected in both cohorts and at least 70% of individuals had observations, resulting in 978 proteins. To increase subgroup sample sizes for different *APOE* alleles, we pooled data from the cohorts. Prior to pooling, we assessed the comparability of the cohorts by examining the distribution and effect size of key protein SMOC1 between controls versus MCI A+. We observed large and comparable effect sizes in both cohorts, indicating that the effects were consistent across datasets. Finally, to reduce the potential influence of outliers on our results in our secondary analysis, we applied winsorization to adjust outlying values (i.e., below the first quartile—1.5 x interquartile range [IQR] and higher than the third quartile + 1.5 x IQR) to those limits. Proteins were annotated to specific brain cell types based on the RNAseq Barres database.^30^

### 2.4 Statistical analysis

Clinical characteristics were compared between *APOE* groups with chi‐squared tests, *t* tests, and one‐way analysis of variance where applicable. For our main analysis, we compared CSF protein levels for each *APOE* genotype to *APOE* ε3/ε3 controls, separately for CN A+ and MCI A+, with linear regression models. We performed additional sensitivity analysis to understand potential influence of disease stage on results by comparing CN A+ ε2/ε3 and ε4 groups to CN A+ ε3/ε3, and MCI A+ ε4 groups to MCI A+ ε3/ε3. We also repeated analyses taking *APOE* genotype as a continuous variable to increase power to detect linear effects. All analyses were adjusted for age, sex, and cohort. All proteins were analyzed in separate models. Statistical analyses were performed with R version 4.2.1—“Funny‐looking Kid” in R studio.^31^

### 2.5 Pathway enrichment analyses

We performed biological pathway enrichment analyses on proteins that were associated with each *APOE* genotype with the Gene Ontology (GO) database, as accessed with PANTHER (version 17.0),^32^, ^33^ and with Elsevier Pathway Collection, as accessed with Enrichr.^34^, ^35^ We used a liberal significance threshold for proteins to ensure that if multiple *APOE*‐associated proteins would be involved in a similar processes that the enrichment analysis would pick this up. We further restricted analyses to a minimum of 10 proteins and used false discovery rate (FDR) adjustment for multiple testing for pathway enrichment *p* values. Enrichment analyses were performed separately for each set of proteins associated with a specific *APOE* genotype in a specific clinical stage and separately for protein levels that were increased versus decreased compared to the ε3/ε3 control group and with the ε3/ε3 group in their own cognitive group. We further annotated proteins as indicative of blood–brain barrier (BBB) dysfunction according to Dayon et al.,^36^ who identified proteins with altered CSF/plasma ratios that were strongly related to the CSF/plasma ratio of albumin as a measure for BBB integrity. As our study only includes CSF measurements, we used their protein list as a proxy to identify potential markers of BBB dysfunction detectable in CSF alone.

## 3 RESULTS

### 3.1 Cohort demographics

In total, 227 CN controls, consisting of 21 ɛ2/ɛ3 (9.3%), 143 ɛ3/ɛ3 (63%), 6 ɛ2/ɛ4 (2.6%), 53 ɛ3/ɛ4 (23.3%), and 4 ɛ4/ɛ4 individuals (1.8%); 165 CN A+ individuals with 11 ɛ2/ɛ3 (6.7%), 49 ɛ3/ɛ3 (29.7%), 4 ɛ2/ɛ4 (2.4%), 79 ɛ3/ɛ4 (47.9%), and 22 ɛ4/ɛ4 (13.3%); and 177 MCI A+ individuals, including 4 ɛ2/ɛ3 (2.3%), 48 ɛ3/ɛ3 (27.1%), 2 ɛ2/ɛ4 (1.1%), 82 ɛ3/ɛ4 (46.3%), and 41 ɛ4/ɛ4 (23.2%), were included (Table S1 describes characteristics for all groups). Due to the limited sample size, we excluded all individuals with *APOE* ε2/ε4 and MCI A+ individuals with *APOE* ε2/ε3 in the main analyses. Table 1 describes the clinical characteristics of the *APOE* genotypes included in the main analysis.

**TABLE 1:** Demographics according to *APOE* genotype and clinical stage for main analyses.

	CN A–T– controls			CN A+				MCI A+			
	ɛ2/ɛ3	ɛ3/ɛ3	ɛ3/ɛ4	ɛ4/ɛ4	ɛ2/ɛ3	ɛ3/ɛ3	ɛ3/ɛ4	ɛ4/ɛ4	ɛ3/ɛ3	ɛ3/ɛ4	ɛ4/ɛ4
N	21	143	53	4	11	49	79	22	48	82	41
Age	59.6 [8.8]	62.7 [7.6]	59.9 [7.5]	59.5 [5.5]	67.8 [8.4]	67.7 [8.4]	66.6 [8.2]	65.1 [7.8]	69.7 [8.4]	68.3 [7.4]	65.8 [5.8]
Sex (female)	11 (52)	65 (46)	17 (32)	1 (25)	6 (55)	30 (61)	43 (54)	12 (55)	20 (42)	42 (51)	16 (39)
Total tau (*Z* score)	0.66 [0.1, 1.2]	0.02 [–0.5, 0.5]	0.13 [–0.5, 0.6]	−0.66 [−1.2, 0.3]	2.98 [0.4, 5.5]^***^ ^,^ ^###^	0.98 [–0.5, 3.1]^**^	1.28 [0.1, 2.4]^***^	0.97 [0, 2.2]^**^	2.18 [0.6, 4.4]^***^	3.50 [1.9,6.2]^***^ ^,^ ^##^	3.10 [2.5, 4.8]^***^ ^#^
AT profile											
A–T–	21 (100)	143 (100)	53 (100)	4 (100)	n/a	n/a	n/a	n/a	n/a	n/a	n/a
A+T–	n/a	n/a	n/a	n/a	5 (46)	29 (63)	47 (67)	15 (68)	22 (46)	22 (27)	5 (12)
A+T+	n/a	n/a	n/a	n/a	6 (56)	17 (37)	23 (33)	7 (32)	26 (54)	60 (73)	36 (88)

*Note*: Results presented in mean [SD] or *n* (%). All individuals with *APOE* ε2/ε4 and MCI A+ individuals with *APOE* ε2/ε3 are excluded from the main analysis and table because of the small group sizes (*n* ≤ 6).

Abbreviations: *APOE*, apolipoprotein E; AT, amyloid and tau; CN, cognitively normal; MCI, mild cognitive impairment; *n*, number; SD, standard deviation.

Compared to *APOE* ɛ3/ɛ3 controls:

* *p* value < 0.05,

** *p* value < 0.01,

*** *p* value < 0.001.

Compared to *APOE* ɛ3/ɛ3 in same clinical group:

^#^ *p* value < 0.1,

^##^ *p* value < 0.01,

^###^ *p* value < 0.001.

**FIGURE 1:**
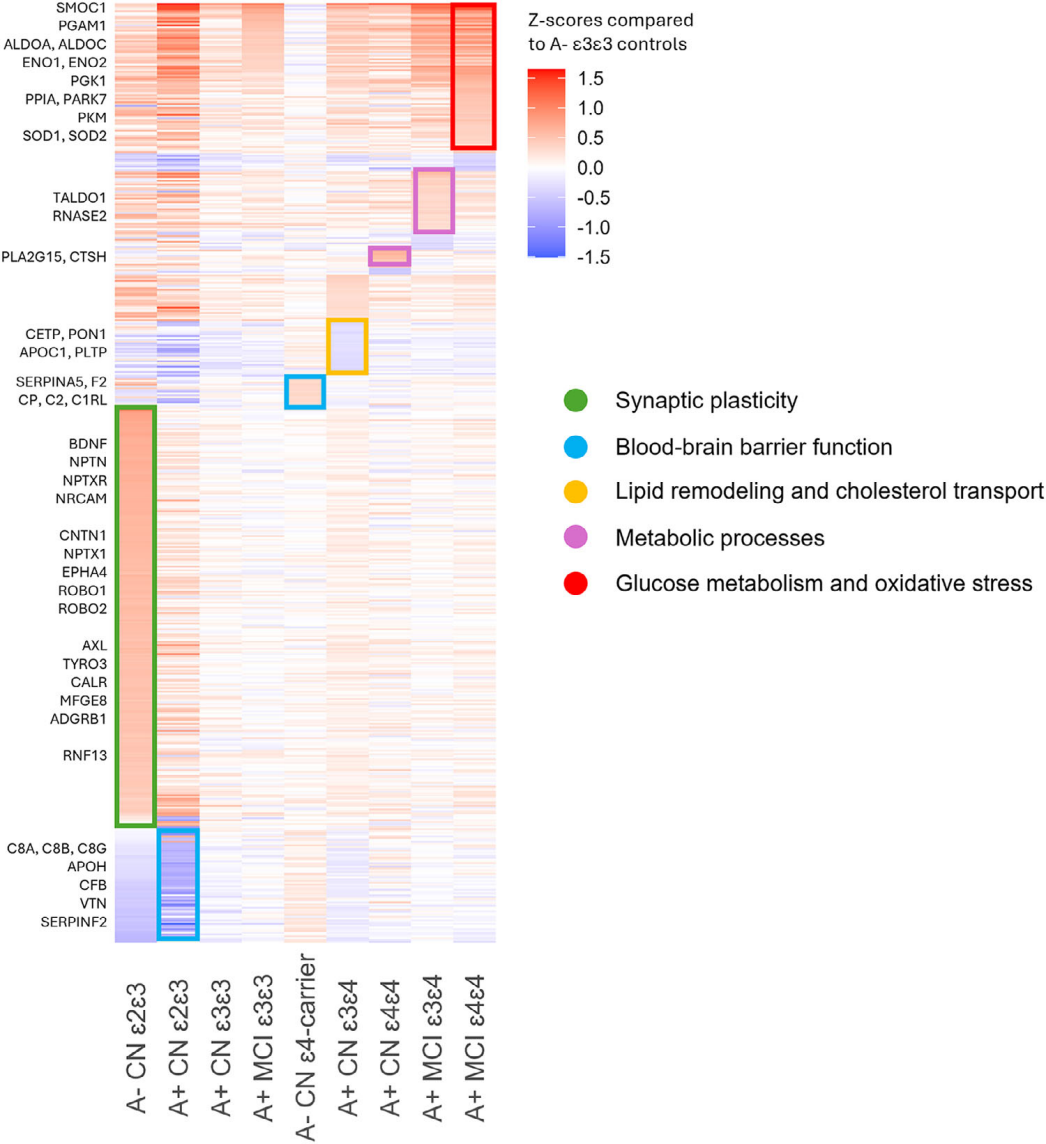
Heatmap of CSF protein levels in each group, compared to ε3/ε3 controls. Red shades indicate higher protein levels, and blue shades indicate lower protein levels compared to controls. CN, cognitively normal; CSF, cerebrospinal fluid; MCI, mild cognitive impairment.

Briefly, A+ individuals were older compared to controls. The distribution of *APOE* genotypes in A+ groups conformed to expectations based on genetic risk, with more ε2 carriers in the amyloid‐negative control group and more ε4 carriers in CN A+ and MCI A+ groups. The proportion of women was somewhat higher in CN A+ compared to controls and MCI A+. MCI A+ had the highest tau levels, whereas controls had the lowest. Furthermore, individuals with more ε4 alleles tended to be younger than individuals without ε4, and in MCI A+, individuals with more ε4 alleles had higher tau levels than individuals without ε4 (Table 1). In the following analyses we compared protein levels in each *APOE* genotype across clinical stages to ε3/ε3 controls and to ε3/ε3 individuals in their own clinical stage. In total, 549/978 (56%) proteins showed differences in one or more *APOE* genotype subgroups compared to our ε3/ε3 control group (Figure 1). Below, we further describe the results separately for ε2 and ε4 compared to ε3/ε3 controls and compared to ε3/ε3 individuals in each clinical group. Detailed results can be found in Tables S2–S5 in supporting information.

### 3.2 Potential mechanisms underlying protective effects of the *APOE* ε2 allele

**FIGURE 2:**
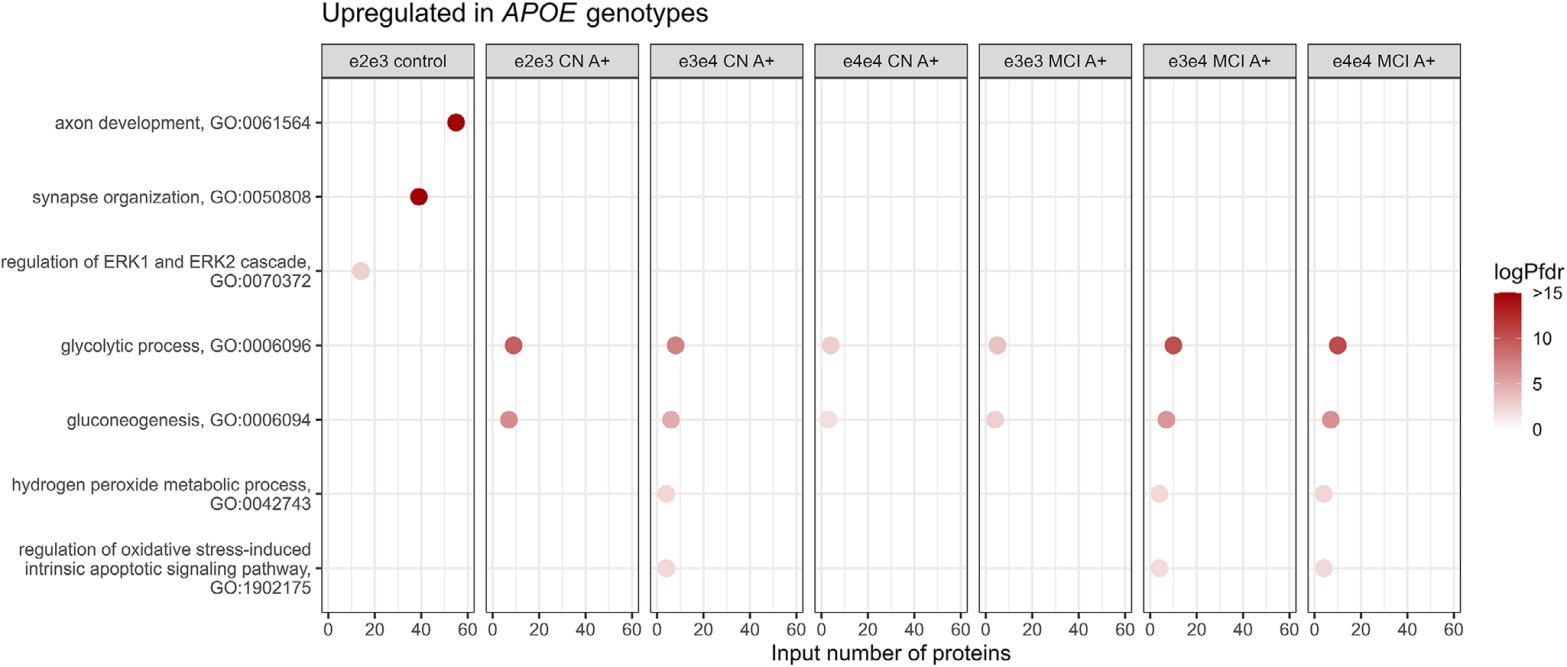
Main GO pathways of proteins upregulated in different *APOE* genotypes across clinical syndromes versus ε3/ε3 controls. From left to right: ε2/ε3 controls, ε2/ε3 CN A+, ε3/ε4 CN A+, ε4/ε4 CN A+, ε3/ε3 MCI A+, ε3/ε4 MCI A+, and ε4/ε4 MCI A+. *APOE*, apolipoprotein E; CN, cognitively normal; GO, Gene Ontology; MCI, mild cognitive impairment.

**FIGURE 3:**
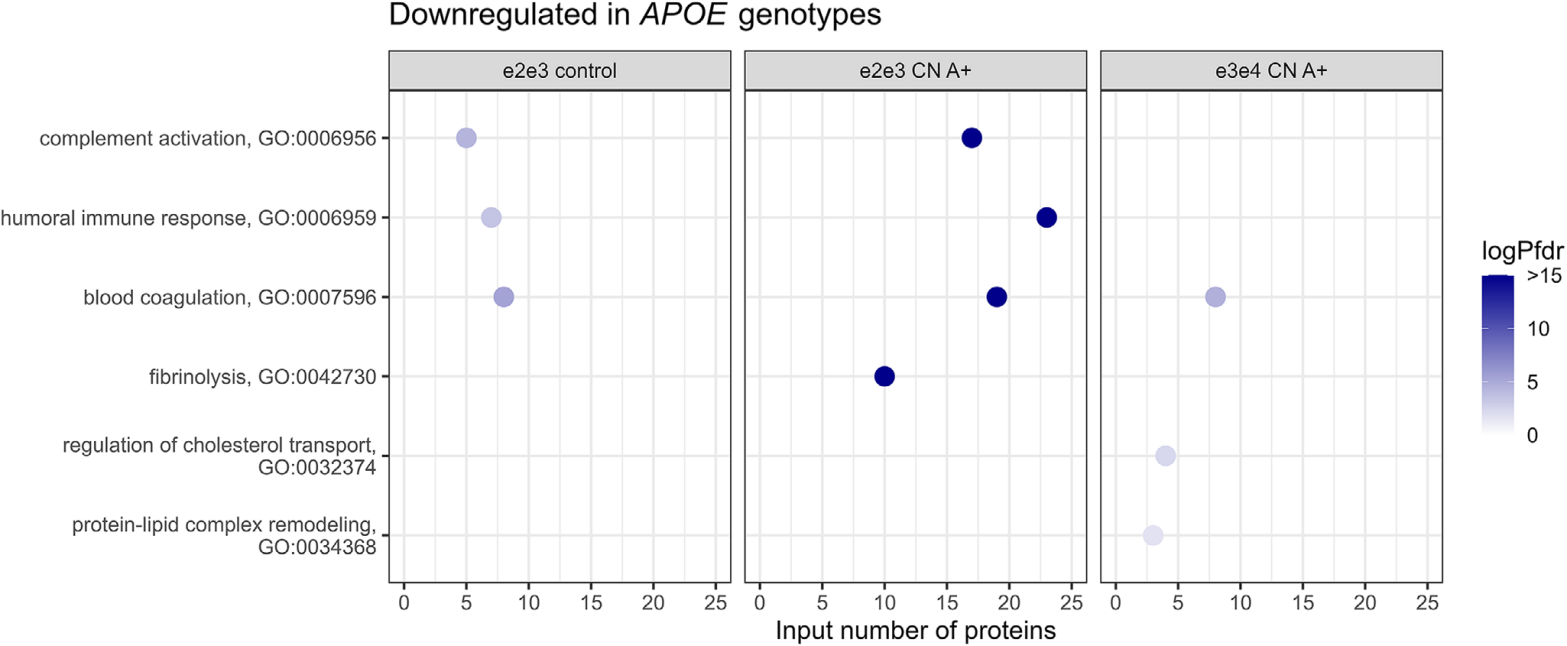
Main GO pathways of proteins downregulated in different APOE genotypes across clinical syndromes versus ε3/ε3 controls. From left to right: ε2/ε3 controls, ε2/ε3 CN A+, and ε3/ε4 CN A+. *APOE*, apolipoprotein E; CN, cognitively normal; GO, Gene Ontology.

We tested CSF proteomic alterations in ε2 controls against ε3/ε3 controls to study which mechanisms may be related to ε2. Of all groups tested, CN A– ε2 carriers had the largest number of proteins with altered levels in CSF, in total 320 (33%), compared to ε3/ε3 controls (Figure 1, Table S2). Most of these proteins (280/320) had increased CSF levels and were enriched for processes related to synaptic plasticity, such as nervous system development (*P*FDR 4.63E‐30), axonogenesis (*P*FDR 1.43E‐15, term = 2), synapse organization (*P*FDR 1.80E‐06), and regulation of the ERK1 and ERK2 cascade (*P*FDR 1.79E‐03), and included proteins such as NRCAM, NPTX1, NPTXR, BDNF, CNTN1, EPHA4, and ROBO1 (Figure 2, Table S4). Many of these proteins were associated with neuronal expression (Figure S1 in supporting information). The proteins in this group were further enriched for the “Eat me signal: apoptotic cell initiates phagocytosis” (*P*FDR 0.002; AXL, TYRO3, CALR, MFGE8, ADGRB1; Table S5) and contained other specific proteins involved in protein degradation, such as RNF13 and BLMH. The 40 proteins with lower CSF levels in ε2/ε3 controls were associated with processes such as coagulation (*P*FDR 4.39E‐06), complement activation (*P*FDR 4.33E‐05), and the humoral immune response (*P*FDR 3.75E‐04), including proteins such as SERPIN‐family members, APOH, F9, C8G, C8B, C8A, CFI, CFB, VTN, and AHSG (Figure 3, Table S4). These proteins were enriched for BBB function (23 out of 73 BBB proteins, *p* value < 0.001). While previous studies indicated higher levels of these proteins are associated with BBB dysfunction,^27^, ^29^ we observe in this ε2 group lower levels of these proteins.

In our combined sample, there were still 11 CN A+ individuals with the ε2/ε3 genotype. Exploratory analysis in this group indicated that compared to ε3/ε3 controls, 79 proteins had higher CSF levels, and these proteins were associated with glucose metabolism (*P*FDR 4.46E‐07, e.g., PKM, TPI1, GOT1, MDH2, PGAM1, PGK1, ENO1, ENO2, GADPH; Figure 2). For 72 proteins the CSF levels were lower than ε3/ε3 controls, which overlapped with proteins decreased in ε2/ε3 controls (22/40; 55%), and were related to BBB function (44 out of 73 BBB proteins, *p* value < 0.001), complement activation (*P*FDR 6.62E‐29), coagulation (*P*FDR 3.33E‐19), and humoral immune response (*P*FDR 6.86E‐22; Figure 3, Table S4). Repeating analyses comparing ε2/ε3 CN A+ to ε3/ε3 CN A+ indicated overlap of 34/79 proteins (43%) with increased levels and 39/72 (54%) with lower levels. Specifically, proteins with lower levels in ε2/ε3 CN A+ compared to ε3/ε3 CN A+ were similarly enriched for complement activation (*P*FDR 1.35E‐19), coagulation (*P*FDR 9.46E‐12), and humoral immune response (*P*FDR 1.51E‐19), indicating that these may be indeed *APOE* ε2 specific, rather than related to amyloid pathology.

### 3.3 Pre‐amyloid changes in controls carrying the *APOE* ε4 allele

Next, we investigated if pre‐amyloid–related alterations could be observed in controls with one or two *APOE* ε4 alleles (*n* = 57). Compared to ε3/ε3 controls, we observed higher levels of 16 and lower levels of 3 proteins in *APOE* ε3/ε4 carriers (*n* = 53). Increased proteins were associated with BBB impairment (4 out of 73 BBB proteins, *P* = 0.03; C2, CP, C1RL, and SERPINA5) and complement activation (*P*FDR = 0.04, including IGLV7‐46 and IGHA1; Table S2). Most protein effects tended to become stronger with the increasing number of ε4 alleles within controls (Table S3). Including control ε4/ε4 carriers (n = 4) in the analysis therefore resulted in more significant increased proteins (*n* = 23) and BBB protein involvement (F2 and AMBP, 6 out of 73 BBB proteins *p* value = 0.02). These processes may reflect early effects of ε4, potentially upstream from amyloid aggregation.

### 3.4 *APOE* ε4–related proteome changes in CN A+ and MCI A+

We next investigated *APOE* ε4–related changes in CSF protein levels in amyloid‐positive individuals and tested if these alterations depended on clinical stage (i.e., CN A+ vs. MCI A+). All A+ ε4 carrier groups had higher levels of proteins (such as PGAM1, GOT1, PKM, PGK1, ENO1, ENO2, ALDOC, and GAPDH) that were enriched for glucose metabolism (*P*FDR range 2.35E‐08 to 6.68E‐04; Figure 2), and to a lesser extent this effect was also observed in amyloid‐positive ε3/ε3 carriers (Table S4). This effect became stronger in MCI A+ compared to CN A+, and within MCI A+ this effect also tended to become stronger with increasing number of ε4 alleles (PGAM1, PGK1, PKM, ALDOA, GAPDH; Table S3). We further observed higher levels with ε4 genotype in oxidative stress–related proteins (e.g., PARK7, PPIA, SOD1, SOD2), which seemed to coincide with these changes in glucose metabolism. Furthermore, in ε3/ε4 CN A+ individuals we observed 36 proteins with lower levels than ε3/ε3 controls that were associated with lipid remodeling (*P*FDR = 0.02) and cholesterol transport (*P*FDR = 0.005, including e.g., CETP, PON1, APOC1, PLTP; Figure 3, Table S4). Repeating analyses comparing ε3/ε4 and ε4/ε4 *APOE* genotypes to ε3/ε3 individuals within CN A+ and MCI A+ indicated that 12 proteins with lower levels in CN A+ ε3/ε4 were associated with lipid‐related processes and cholesterol transport, similarly compared to controls. Comparing ε4/ε4 MCI A+ individuals to ε3/ε3 MCI A+ indicated that 22 proteins with higher levels were related to metabolic processes (GO pathway *P*FDR range = 0.039 to 0.055; Table S4).

### 3.5 Comparing *APOE*‐related proteome changes across disease stages to autosomal dominant AD and Down syndrome AD

**FIGURE 4:**
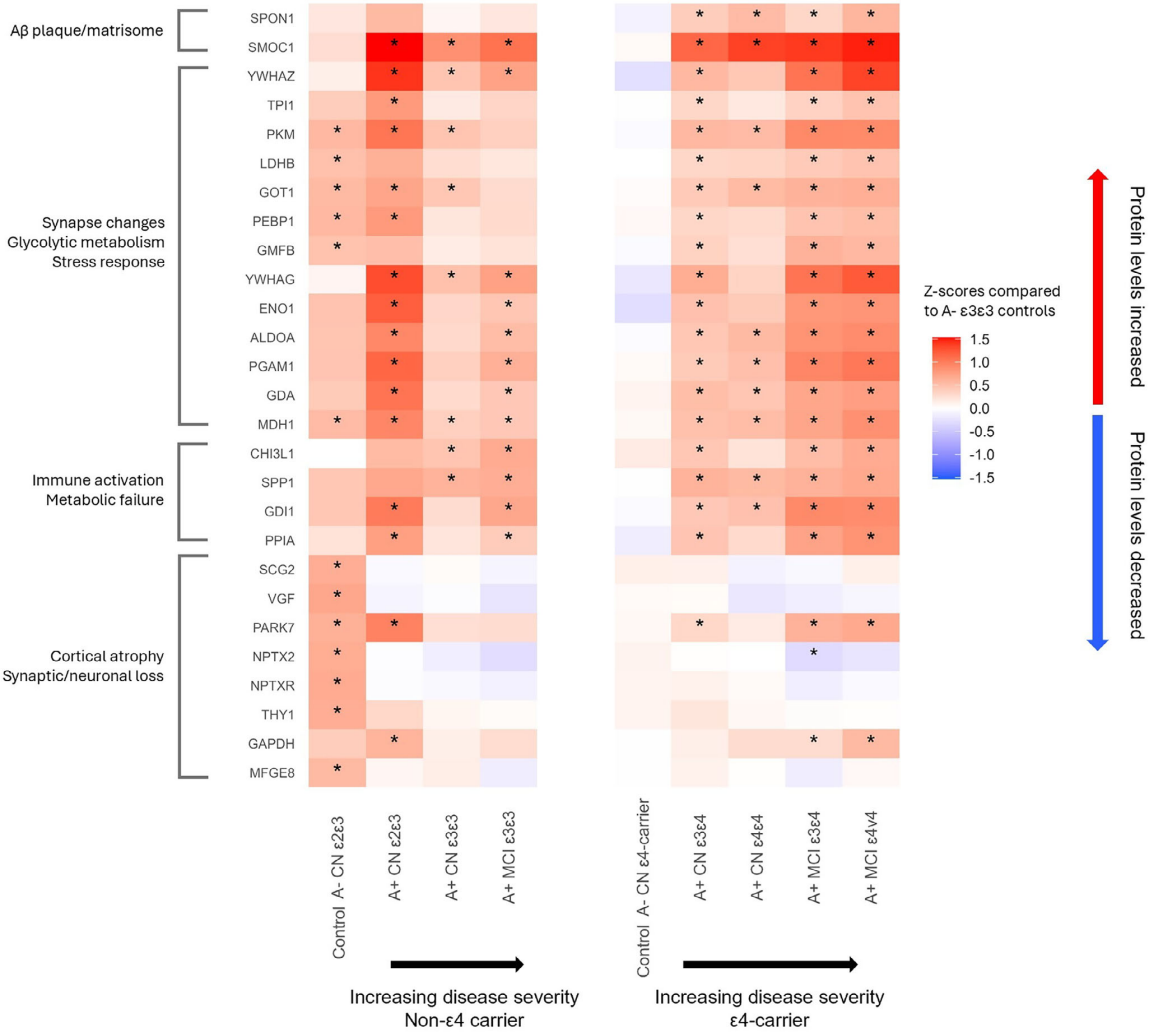
Protein changes stratified by amyloid status, *APOE* genotype, and clinical stage, revealing how molecular changes track the trajectory of sporadic AD, allowing for comparison to autosomal dominant AD and Down syndrome‐associated AD.^37^, ^38^, ^46^ AD, Alzheimer's disease; *APOE*, apolipoprotein E; CN, cognitively normal; MCI, mild cognitive impairment.

Finally, we made a heatmap (Figure 4) for a selection of proteins that have previously been reported to be altered in the natural history of autosomal dominant AD (ADAD)^37^ and in Down syndrome AD (DSAD)^38^ to understand how *APOE*‐related changes in pre‐dementia stage of sporadic AD compare to those previous studies. Figure 4 indicates that SMOC1 was most elevated in A+ individuals with both intact cognition and MCI regardless of *APOE* genotype, which seems consistent with early effects reported in ADAD and DSAD. However, while in ADAD SMOC1 elevations were closely related to decreasing Aβ in the disease course, we did not observe increased SMOC1 levels in *APOE* ε4 A–. Furthermore, we observed that SPON1, which had high levels in ADAD and DSAD in very early amyloid aggregation stages, was only elevated in A+ ε4 carriers in all stages, but not in A+ ε4 non‐carriers. We found proteins related to synapse changes, glucose metabolism, and stress response (e.g., YWHAZ, PKM, YWHAG, ENO1, ALDOA, and PGAM1) that were increased early in ADAD and DSAD and were also elevated in all A+ CN individuals, regardless of *APOE* genotype. While this effect became stronger in MCI A+ compared to CN A+ in ε4 carriers, in non‐carriers the effects were strongest in the CN A+, suggesting a different timing of processes associated with these proteins depending on *APOE* genotype. This seems to be different from the timings of alterations observed in ADAD, in which some of the metabolism‐related proteins (LDHB, GOT1, PEBP1, TPI1, GMFB) were observed to have transient pre‐dementia increases, and others (YWHAZ, PKM, YWHAG, ENO1, PGAM1) remain elevated post‐dementia. In DSAD, by contrast, some of these proteins (ENO1, PEBP1, LDHB, TPI1) were reduced before dementia onset, while others rose much closer to or even post dementia onset. Furthermore, we compared a group of proteins related to synaptic and neurosecretory processes (SCG2, VGF, NPTX2, NPTXR, THY1, MFGE8). While these proteins are decreased after ADAD or before DSAD dementia onset, consistent with synaptic and neuronal loss, we observed elevation of these proteins in our ε2/ε3 CN A– carriers. Finally, we observed that several proteins related to metabolism (PKM, LDHB, GOT1, PEBP1, GMFB, MDH1) that were increased in ADAD and DSAD were also increased in our CN A– ε2/ε3 carriers, which was unexpected as these individuals are least likely to develop AD.

## 4 DISCUSSION

In this study we investigated the effects of *APOE* genotype on the CSF proteome in individuals with normal cognition and normal CSF amyloid and tau, and in individuals with abnormal amyloid without dementia. While the *APOE* gene includes both the strongest risk and protective factors for sporadic AD, little is known about the underlying mechanisms through which these genotypes exert their effects, in particular because only 10% to 15% of the population carries the protective ε2 genotype.^5^, ^6^ By leveraging two large cohorts, we assembled a large enough sample to study this question, using a CSF proteomic approach to probe molecular processes in great detail. We observed the most pronounced effects of *APOE* ε2/ε3 controls compared to ε3/ε3 controls, with protein alterations pointing toward involvement of synaptic plasticity‐related processes, the eat‐me‐signal, and BBB integrity. On the other hand, ε4 carrier controls had CSF protein changes indicative of BBB dysfunction, suggesting that BBB alterations may occur upstream in terms of both protective and risk effects of *APOE* genotype. Furthermore, we observed that protein levels associated with *APOE* ε4 in CN A+ and MCI A+ individuals were associated with glucose metabolism, oxidative stress, and lipid remodeling, suggesting that *APOE* ε4 risk may impact these processes downstream from amyloid pathology. In summary, these results indicate that protective mechanisms of ε2 and risk mechanisms of ε4 affect the CSF proteome, involving both similar and distinct processes.

While a great body of literature on *APOE* exists,^13^, ^39^ few studies so far have investigated associations of *APOE* genotype in patients through CSF proteomic analysis, and all have focused on ε4‐related effects. Two studies using the Alzheimer's Disease Neuroimaging Initiative (ADNI) dataset investigated *APOE* effects in AD, which included panels of 200 and 300 targeted proteins.^10^, ^11^ Two recent studies, within ADNI^40^ and the Global Neurodegeneration Proteomics Consortium cohort,^41^ used the SomaScan platform (> 6000 proteins) and reported, respectively, 57 and 229 proteins related to *APOE* ε4 genotype that were related to innate immune activation, inflammation, and indications of BBB dysfunction in *APOE* ε4 carriers, which we also observe. An ADNI study indicated that *APOE* ε4 was associated with higher injury marker levels such as tau and neurogranin, which we also observed in the current independent cohort.^10^ Also in ADNI, we^10^ and others^11^ reported decreased levels of complement factors and related proteins in ε4 carriers. In this study we replicated some of these findings (e.g., lower levels of KNG1, APCS, and C7 in ε3/ε4 CN A+), and detected four additional proteins (CP, C1RL, SERPINA5, and AMBP) to be altered in ε4 A– controls, pointing toward early BBB disruption. As these proteins were not measured in previous ADNI studies, our untargeted approach might provide insight into potential pre‐amyloid mechanisms of ε4. Furthermore, in contrast to previous findings, we observed increased levels of other proteins (C2 and F2) associated with the complement pathway already in ε4 carrier controls. Possibly, differences in cohort composition (individuals in our study were ≈ 10 years younger compared to ADNI), size, and methodological variations (we used untargeted TMT‐based proteomics, while ADNI relied on targeted mass spectroscopy) may explain these diverging findings.

Another new finding from our study was that because we could pool two large cohorts, we were able to investigate the effect of ε2 on the CSF proteome. As the ε2 allele is only present in 10% to 15% of the population,^5^ our large cohort allowed for unique analysis into the protective biological effects of ε2. In these analyses, we found a large‐scale proteomic signature related to ε2 carrier controls in which proteins had higher levels than in ε3/ε3 controls and were enriched for neuronal plasticity‐related processes. This suggests that possibly one mechanism through which *APOE* ε2 exerts protection against AD may be through neuronal connectivity. Previous animal studies have described a neurotrophic effect of ε2, which helps to preserve neurons and synaptic functions.^13^ Our ε2 controls further had higher levels of proteins associated with protein degradation and the “eat‐me” signal, which is expressed on cells or particles to promote phagocytosis and clearance of, for example, apoptotic cells, which is important for maintaining tissue homeostasis.^42^ Possibly, this may reflect a mechanism through which clearance may be enhanced. Furthermore, we found that proteins of which higher CSF levels have previously been related to (loss of) BBB integrity^27^, ^29^, ^36^ were lower in ε2/ε3 controls as well as ε2/ε3 CN A+ individuals. Possibly, the notion that these same proteins were lower than controls in ε2 may indicate that this genotype also influences BBB integrity, possibly reflecting protective effects. However, large longitudinal studies mapping how amyloid and tau pathology develops in ε2 are necessary to test whether these pathways truly confer protection or risk upstream from amyloid aggregation.

Furthermore, we observed that complement‐related proteins had elevated CSF levels already in control ε4 carriers. Perhaps we could only now detect this effect due to the larger sample size in this study (*n* = 57 ε4 carrier controls, compared to *n* = 8 in previous studies). Amyloid plaques trigger complement activation,^43^ and *APOE* ε4 has been shown to enhance this activation.^44^ Possibly we observe a response to very early amyloid‐related changes in our ε4 carrier controls. Longitudinal studies with repeated CSF measurements are necessary to further investigate this question. Furthermore, we observed downregulation of proteins involved in lipid metabolism in A+ ε4 carriers, both compared to controls and ε3/ε3 CN A+ individuals, suggesting a highly specific *APOE*‐related mechanism. *APOE*’s role in lipid transport is well known, and both impaired lipid transport and poor lipidation in ε4 carriers may further exacerbate *APOE*‐related detrimental processes (e.g., less efficient amyloid clearance, decreased neurotrophic functioning) in AD.^9^ A previous experimental study in mice reported that microglia activation in the presence of amyloid aggregation differed in *APOE* ε4 lipoprotein composition compared to ε3, resulting in restricted surrounding of microglia around plaques and reduced Aβ uptake.^45^ It would be of interest to further investigate the *APOE* genotype with a combined proteomics and lipidomics study in CSF in patients.

Finally, we used our stratification by amyloid status, *APOE* genotype, and clinical diagnosis as a cross‐sectional approximation for disease trajectories, comparing *APOE*‐related proteins in sporadic AD to those reported in ADAD and DSAD.^37^, ^38^, ^46^ In those studies, SMOC1 was elevated very early, and we similarly found increased SMOC1 as the strongest effect of A+ across all groups, supporting robust early involvement in amyloid aggregation independent of *APOE*. SPON1, an extracellular matrix protein elevated early in ADAD and DSAD, was increased only in *APOE* A+ ε4 carriers, suggesting a genotype‐dependent effect. Notably, a subset of metabolic and synaptic integrity proteins, reported to be altered in ADAD and DSAD, were also increased in our CN A– ε2/ε3 carriers, suggesting these may also underlie processes of resilience. Future research is needed to understand whether this signature indeed contributes to reduced cognitive decline.

Some limitations have to be noted about this study. First, although our overall group size was large because we combined two cohorts, the number of individuals with rare *APOE* genotypes remained relatively small, especially ε2 A+ MCI individuals (reflecting its protective effects) and homozygous ε4 controls (reflecting its risk effects). Therefore, we were unable to specifically study these subgroups, and we aim to increase our sample sizes for future research. This also limited our ability to further subdivide groups into different amyloid and tau biomarker subgroups. Still, using tau as a continuous outcome, *APOE* ε4 genotype was associated with higher t‐tau levels, particularly in later clinical stages. Larger studies are needed to further investigate if *APOE* genotypes in combination with amyloid and tau biomarker profiles may be related to specific proteomic signatures. Second, CSF proteomics do not directly reveal the anatomical origin of proteomic alterations, leaving it unclear whether CSF proteomic concentrations may reflect better global or regional BBB integrity in ε2/ε3 individuals. Imaging approaches such as arterial spin labelling perfusion magnetic resonance imaging are required to spatially map BBB integrity, and studies within the Developing BBB‐ASL as a Non‐Invasive Early Biomarker (DEBBIE) consortium are currently addressing this.^47^ In addition, new experimental BBB models, such as induced pluripotent stem cell–derived systems with different *APOE* genotypes, may provide complementary mechanistic insights into the genotype‐specific CSF protein changes. Third, our analyses were cross‐sectional, and so the differences between clinical stages we observed require further verification with longitudinal proteomic data to understand how processes change with disease progression. Finally, while our findings may point toward processes that could play a role in disease pathogenesis through CSF proteomics, functional follow‐up studies are necessary to further study mechanisms. A strength of our study was the large overall sample size, enabling us to characterize CSF proteomic alterations related to ε2, which has not been studied before in such detail in humans. In addition, the use of untargeted proteomics allowed for identifying biological processes associated with *APOE* genotype in A+ individuals and controls, which were not captured in previous CSF proteomic studies using a targeted approach.

In conclusion, we completed one of the largest studies investigating effects of *APOE* genotype on the CSF proteome in older individuals with and without AD pathology, which provides novel insights into the protective mechanisms of *APOE* ε2 and risk mechanisms of *APOE* ε4.

## CONFLICT OF INTEREST STATEMENT

J.P. received consultation and speaker honoraria from Nestle Institute of Health Sciences, Innovation Campus, EPFL, Lausanne, Switzerland; Ono Pharma, Schwabe Pharma Switzerland; OM Pharma Switzerland; Roche Pharma; and Fujirebio Europe, all not related to this work. H.Z. has served on scientific advisory boards and/or as a consultant for AbbVie, Acumen, Alector, Alzinova, ALZPath, Annexon, Apellis, Artery Therapeutics, AZTherapies, Cognito Therapeutics, CogRx, Denali, Eisai, Nervgen, Novo Nordisk, Optoceutics, Passage Bio, Pinteon Therapeutics, Prothena, Red Abbey Labs, reMYND, Roche, Samumed, Siemens Healthineers, Triplet Therapeutics, and Wave; has given lectures in symposia sponsored by Alzecure, Biogen, Cellectricon, Fujirebio, Lilly, and Roche; and is a co‐founder of Brain Biomarker Solutions in Gothenburg AB (BBS), which is a part of the GU Ventures Incubator Program (outside submitted work). C.E.T. is employed by Amsterdam UMC. She has grants or contracts for Research of the European Commission (Marie Curie International Training Network, grant agreement No 860197 (MIRIADE); Innovative Medicines Initiatives 3TR (Horizon 2020, grant no. 831434), EPND (IMI 2 Joint Undertaking [JU], grant No. 101034344) and JPND (bPRIDE), National MS Society (Progressive MS Alliance), Alzheimer Drug Discovery Foundation, Alzheimer Association, Health Holland, and the Dutch Research Council (ZonMW), including TAP‐dementia, a ZonMw‐funded project (#10510032120003) in the context of the Dutch National Dementia Strategy, Alzheimer Drug Discovery Foundation, the Selfridges Group Foundation, and Alzheimer Netherlands. She is the recipient of ABOARD, which is a public–private partnership receiving funding from ZonMW (#73305095007) and Health∼Holland, Topsector Life Sciences & Health (PPP‐allowance; #LSHM20106). She is also a contract researcher for ADx Neurosciences, AC‐Immune, Aribio, Axon Neurosciences, Beckman‐Coulter, BioConnect, Bioorchestra, Brainstorm Therapeutics, Celgene, Cognition Therapeutics, EIP Pharma, Eisai, Eli Lilly, Fujirebio, Grifols, Instant Nano Biosensors, Merck, Novo Nordisk, Olink, PeopleBio, Quanterix, Roche, Siemens, Toyama, Vivoryon, and the European Commission. She has received payment or honoraria from Roche, Novo Nordisk, and Grifols, where all payments were made to her institution. She also serves on editorial boards of Medidact Neurologie/Springer; and in *Neurology: Neuroimmunology & Neuroinflammation*. She is editor of *Alzheimer Research and Therapy*. R.V.’s institution has clinical trial agreements (R.V. as PI) with Alector, AviadoBio, Biogen, Denali, J&J, and UCB. R.V.’s institution has consultancy agreements (R.V. as DSMB chair) with AC Immune and with Eli Lilly and Roche (R.V. as member of the advisory board). K.B. has served as a consultant and on advisory boards for Acumen, ALZPath, BioArctic, Biogen, Eisai, Lilly, Moleac Pte. Ltd, Novartis, Ono Pharma, Prothena, Roche Diagnostics, and Siemens Healthineers; has served on data monitoring committees for Julius Clinical and Novartis; has given lectures, produced educational materials, and participated in educational programs for AC Immune, Biogen, Celdara Medical, Eisai, and Roche Diagnostics; and is a co‐founder of Brain Biomarker Solutions in Gothenburg AB (BBS), which is a part of the GU Ventures Incubator Program, outside the work presented in this paper. P.S. is a full‐time employee of EQT Life Sciences (formerly LSP) and professor emeritus at Amsterdam University Medical Centers. He has received consultancy fees (paid to the university) from Alzheon, Brainstorm Cell, and Green Valley. Within his university affiliation, he was global PI of the phase 1b study of AC Immune, phase 2b study with FUJI‐film/Toyama, and the phase 2 study of UCB. He is past chair of the EU steering committee of the phase 2b program of Vivoryon and the phase 2b study of Novartis Cardiology and presently co‐chair of the phase 3 study with NOVO‐Nordisk. P.J.V. has received honoraria for a workshop on grant writing organized by Stiftung Synapsis, Alzheimer Forschung Schweiz AFS (payment to university), and is a member of the executive board of EADC. P.J.V. and B.M.T. have a patent on AD subtypes (PCT/NL2020/050216). The remaining authors report no conflicts of interest. Author disclosures are available in the supporting information.

## CONSENT STATEMENT AND ETHICS APPROVAL

Written informed consent was obtained from all participants or surrogates. The medical ethics committees at each study site approved the studies.

## Supporting information



Supporting Information

Supporting Information

Supporting Information

## References

[1] Corder EH , Saunders AM , Strittmatter WJ , et al. Gene dose of apolipoprotein E type 4 allele and the risk of Alzheimer's disease in late onset families. Science. 1993;261(5123):921‐923. 8346443 10.1126/science.8346443

[2] Saunders AM , Strittmatter WJ , Schmechel D , et al. Association of apolipoprotein E allele epsilon 4 with late‐onset familial and sporadic Alzheimer's disease. Neurology. 1993;43(8):1467‐1472. 8350998 10.1212/wnl.43.8.1467

[3] Fortea J , Pegueroles J , Alcolea D , et al. Publisher correction: aPOE4 homozygosity represents a distinct genetic form of Alzheimer's disease. Nat Med. 2024;30(7):2093. 10.1038/s41591-024-03127-y 38886628

[4] Jansen WJ , Ossenkoppele R , Knol DL , et al. Prevalence of cerebral amyloid pathology in persons without dementia: a meta‐analysis. Jama. 2015;313(19):1924‐1938. 25988462 10.1001/jama.2015.4668 PMC4486209

[5] Kim H , Devanand DP , Carlson S , Goldberg TE . Apolipoprotein E genotype e2: neuroprotection and its limits. Front Aging Neurosci. 2022;14:919712. 35912085 10.3389/fnagi.2022.919712 PMC9329577

[6] Belloy ME , Andrews SJ , Le Guen Y , et al. APOE genotype and Alzheimer disease risk across age, sex, and population ancestry. JAMA Neurol. 2023;80(12):1284‐1294. 37930705 10.1001/jamaneurol.2023.3599 PMC10628838

[7] Rajan KB , Barnes LL , Wilson RS , et al. Racial differences in the association between apolipoprotein E risk alleles and overall and total cardiovascular mortality over 18 years. J Am Geriatr Soc. 2017;65(11):2425‐2430. 28898389 10.1111/jgs.15059 PMC6201232

[8] Holtzman DM , Herz J , Bu G . Apolipoprotein E and apolipoprotein E receptors: normal biology and roles in Alzheimer disease. Cold Spring Harb Perspect Med. 2012;2(3):a006312. 22393530 10.1101/cshperspect.a006312 PMC3282491

[9] Yamazaki Y , Zhao N , Caulfield TR , Liu CC , Bu G . Apolipoprotein E and Alzheimer disease: pathobiology and targeting strategies. Nat Rev Neurol. 2019;15(9):501‐518. 31367008 10.1038/s41582-019-0228-7 PMC7055192

[10] Konijnenberg E , Tijms BM , Gobom J , et al. APOE ε4 genotype‐dependent cerebrospinal fluid proteomic signatures in Alzheimer's disease. Alzheimers Res Ther. 2020;12(1):65. 32460813 10.1186/s13195-020-00628-z PMC7254647

[11] Berger M , Cooter M , Roesler AS , et al. Erratum to: aPOE4 copy number‐dependent proteomic changes in the cerebrospinal fluid. J Alzheimers Dis. 2022;90(3):1339‐1340. 36373324 10.3233/JAD-229018 PMC9756142

[12] Lumsden AL , Mulugeta A , Zhou A , Hyppönen E . Apolipoprotein E (APOE) genotype‐associated disease risks: a phenome‐wide, registry‐based, case‐control study utilising the UK Biobank. EBioMedicine. 2020;59:102954. 32818802 10.1016/j.ebiom.2020.102954 PMC7452404

[13] Li Z , Shue F , Zhao N , Shinohara M , Bu G . APOE2: protective mechanism and therapeutic implications for Alzheimer's disease. Mol Neurodegener. 2020;15(1):63. 33148290 10.1186/s13024-020-00413-4 PMC7640652

[14] Conejero‐Goldberg C , Gomar JJ , Bobes‐Bascaran T , et al. APOE2 enhances neuroprotection against Alzheimer's disease through multiple molecular mechanisms. Mol Psychiatry. 2014;19(11):1243‐1250. 24492349 10.1038/mp.2013.194

[15] Chung WS , Verghese PB , Chakraborty C , et al. Novel allele‐dependent role for APOE in controlling the rate of synapse pruning by astrocytes. Proc Natl Acad Sci USA. 2016;113(36):10186‐10191. 27559087 10.1073/pnas.1609896113 PMC5018780

[16] van der Flier WM , Scheltens P . Amsterdam dementia cohort: performing research to optimize care. J Alzheimers Dis. 2018;62(3):1091‐1111. 29562540 10.3233/JAD-170850 PMC5870023

[17] Bos I , Vos S , Vandenberghe R , et al. The EMIF‐AD multimodal biomarker discovery study: design, methods and cohort characteristics. Alzheimers Res Ther. 2018;10(1):64. 29980228 10.1186/s13195-018-0396-5 PMC6035398

[18] Petersen RC , Smith GE , Waring SC , Ivnik RJ , Tangalos EG , Kokmen E . Mild cognitive impairment: clinical characterization and outcome. Arch Neurol. 1999;56(3):303‐308. 10190820 10.1001/archneur.56.3.303

[19] Winblad B , Palmer K , Kivipelto M , et al. Mild cognitive impairment–beyond controversies, towards a consensus: report of the International Working Group on mild cognitive impairment. J Intern Med. 2004;256(3):240‐246. 15324367 10.1111/j.1365-2796.2004.01380.x

[20] Albert MS , DeKosky ST , Dickson D , et al. The diagnosis of mild cognitive impairment due to Alzheimer's disease: recommendations from the National Institute on Aging‐Alzheimer's Association workgroups on diagnostic guidelines for Alzheimer's disease. Alzheimers Dement. 2011;7(3):270‐279. 21514249 10.1016/j.jalz.2011.03.008 PMC3312027

[21] van der Flier WM , Pijnenburg YA , Prins N , et al. Optimizing patient care and research: the Amsterdam dementia cohort. J Alzheimers Dis. 2014;41(1):313‐327. 24614907 10.3233/JAD-132306

[22] Bos I , Vos S , Verhey F , et al. Cerebrospinal fluid biomarkers of neurodegeneration, synaptic integrity, and astroglial activation across the clinical Alzheimer's disease spectrum. Alzheimers Dement. 2019;15(5):644‐654. 30853464 10.1016/j.jalz.2019.01.004

[23] Engelborghs S , Niemantsverdriet E , Struyfs H , et al. Consensus guidelines for lumbar puncture in patients with neurological diseases. Alzheimers Dement (Amst). 2017;8:111‐126. 28603768 10.1016/j.dadm.2017.04.007 PMC5454085

[24] Hok‐A‐Hin YS , Willemse EAJ , Teunissen CE , Del Campo M . Guidelines for CSF processing and biobanking: impact on the Identification and Development of Optimal CSF Protein Biomarkers. Methods Mol Biol. 2019;2044:27‐50. 31432404 10.1007/978-1-4939-9706-0_2

[25] Mulder C , Verwey NA , van der Flier WM , et al. Amyloid‐beta(1‐42), total tau, and phosphorylated tau as cerebrospinal fluid biomarkers for the diagnosis of Alzheimer disease. Clin Chem. 2010;56(2):248‐253. 19833838 10.1373/clinchem.2009.130518

[26] Tijms BM , Willemse EAJ , Zwan MD , et al. Unbiased approach to counteract upward drift in cerebrospinal fluid amyloid‐beta 1‐42 analysis results. Clin Chem. 2018;64(3):576‐585. 29208658 10.1373/clinchem.2017.281055

[27] Tijms BM , Gobom J , Reus L , et al. Pathophysiological subtypes of Alzheimer's disease based on cerebrospinal fluid proteomics. Brain. 2020;143(12):3776‐3792. 33439986 10.1093/brain/awaa325 PMC7805814

[28] Plubell DL , Wilmarth PA , Zhao Y , et al. Extended multiplexing of tandem mass tags (TMT) labeling reveals age and high fat diet specific proteome changes in mouse epididymal adipose tissue. Mol Cell Proteomics. 2017;16(5):873‐890. 28325852 10.1074/mcp.M116.065524 PMC5417827

[29] Tijms BM , Vromen EM , Mjaavatten O , et al. Cerebrospinal fluid proteomics in patients with Alzheimer's disease reveals five molecular subtypes with distinct genetic risk profiles. Nat Aging. 2024;4(1):33‐47. 38195725 10.1038/s43587-023-00550-7 PMC10798889

[30] Zhang Y , Chen K , Sloan SA , et al. An RNA‐sequencing transcriptome and splicing database of glia, neurons, and vascular cells of the cerebral cortex. J Neurosci. 2014;34(36):11929‐11947. 25186741 10.1523/JNEUROSCI.1860-14.2014 PMC4152602

[31] R Core Team . R: a language and environment for statistical computing. R Foundation for Statistical Computing, 2019. https://www.R-project.org/

[32] Mi H , Muruganujan A , Ebert D , Huang X , Thomas PD . PANTHER version 14: more genomes, a new PANTHER GO‐slim and improvements in enrichment analysis tools. Nucleic Acids Res. 2019;47(D1):D419‐d26. 30407594 10.1093/nar/gky1038 PMC6323939

[33] Thomas PD , Ebert D , Muruganujan A , Mushayahama T , Albou LP , Mi H . PANTHER: making genome‐scale phylogenetics accessible to all. Protein Sci. 2022;31(1):8‐22. 34717010 10.1002/pro.4218 PMC8740835

[34] Chen EY , Tan CM , Kou Y , et al. Enrichr: interactive and collaborative HTML5 gene list enrichment analysis tool. BMC Bioinformatics. 2013;14:128. 23586463 10.1186/1471-2105-14-128 PMC3637064

[35] Kuleshov MV , Jones MR , Rouillard AD , et al. Enrichr: a comprehensive gene set enrichment analysis web server 2016 update. Nucleic Acids Res. 2016;44(W1):W90‐W97. 27141961 10.1093/nar/gkw377 PMC4987924

[36] Dayon L , Cominetti O , Wojcik J , et al. Proteomes of paired human cerebrospinal fluid and plasma: relation to blood‐brain barrier permeability in older adults. J Proteome Res. 2019;18(3):1162‐1174. 30702894 10.1021/acs.jproteome.8b00809

[37] Johnson ECB , Bian S , Haque RU , et al. Cerebrospinal fluid proteomics define the natural history of autosomal dominant Alzheimer's disease. Nat Med. 2023;29(8):1979‐1988. 37550416 10.1038/s41591-023-02476-4 PMC10427428

[38] Montoliu‐Gaya L , Bian S , Dammer EB , et al. Proteomic analysis of Down syndrome cerebrospinal fluid compared to late‐onset and autosomal dominant Alzheimer s disease. Nat Commun. 2025;16(1):6003. 40595720 10.1038/s41467-025-61054-z PMC12214755

[39] Serrano‐Pozo A , Das S , Hyman BT . APOE and Alzheimer's disease: advances in genetics, pathophysiology, and therapeutic approaches. Lancet Neurol. 2021;20(1):68‐80. 33340485 10.1016/S1474-4422(20)30412-9 PMC8096522

[40] Shvetcov A , Thomson S , Cho AN , et al. Proteome profiling of cerebrospinal fluid using machine learning shows a unique protein signature associated with APOE4 genotype. Aging Cell. 2025;24(4):e14439. 39722190 10.1111/acel.14439 PMC11984689

[41] Shvetcov A , Johnson ECB , Winchester LM , et al. APOE epsilon4 carriers share immune‐related proteomic changes across neurodegenerative diseases. Nat Med. 2025;31(8):2590‐2601. 40665049 10.1038/s41591-025-03835-z PMC12353839

[42] Bagalkot V , Deiuliis JA , Rajagopalan S , Maiseyeu A . Eat me″ imaging and therapy. Adv Drug Deliv Rev. 2016;99:2‐11. Pt A. 26826436 10.1016/j.addr.2016.01.009 PMC4865253

[43] Veerhuis R , Nielsen HM , Tenner AJ . Complement in the brain. Mol Immunol. 2011;48(14):1592‐1603. 21546088 10.1016/j.molimm.2011.04.003 PMC3142281

[44] McGeer PL , Walker DG , Pitas RE , Mahley RW , McGeer EG . Apolipoprotein E4 (ApoE4) but not ApoE3 or ApoE2 potentiates beta‐amyloid protein activation of complement in vitro. Brain Res. 1997;749(1):135‐138. 9070638 10.1016/s0006-8993(96)01324-8

[45] Fitz NF , Nam KN , Wolfe CM , et al. Phospholipids of APOE lipoproteins activate microglia in an isoform‐specific manner in preclinical models of Alzheimer's disease. Nat Commun. 2021;12(1):3416. 34099706 10.1038/s41467-021-23762-0 PMC8184801

[46] Higginbotham L , Ping L , Dammer EB , et al. Integrated proteomics reveals brain‐based cerebrospinal fluid biomarkers in asymptomatic and symptomatic Alzheimer's disease. Sci Adv. 2020;6(43):eaaz9360. 33087358 10.1126/sciadv.aaz9360 PMC7577712

[47] Padrela B , Mahroo A , Tee M , et al. Developing blood‐brain barrier arterial spin labelling as a non‐invasive early biomarker of Alzheimer's disease (DEBBIE‐AD): a prospective observational multicohort study protocol. BMJ Open. 2024;14(3):e081635. 10.1136/bmjopen-2023-081635 PMC10928768 38458785

