# Supplementary material for: Cerebrospinal fluid proteomic associations of APOE genotypes reveal distinct protective and risk mechanisms for Alzheimer's disease
Source: Alzheimers Dement. 2025 Oct 14;21(10):e70738. doi: 10.1002/alz.70738 (PMC12519502; doi:10.1002/alz.70738)
Supplement: Supplementary file 1 — Supporting Information [file ALZ-21-e70738-s002.pdf]

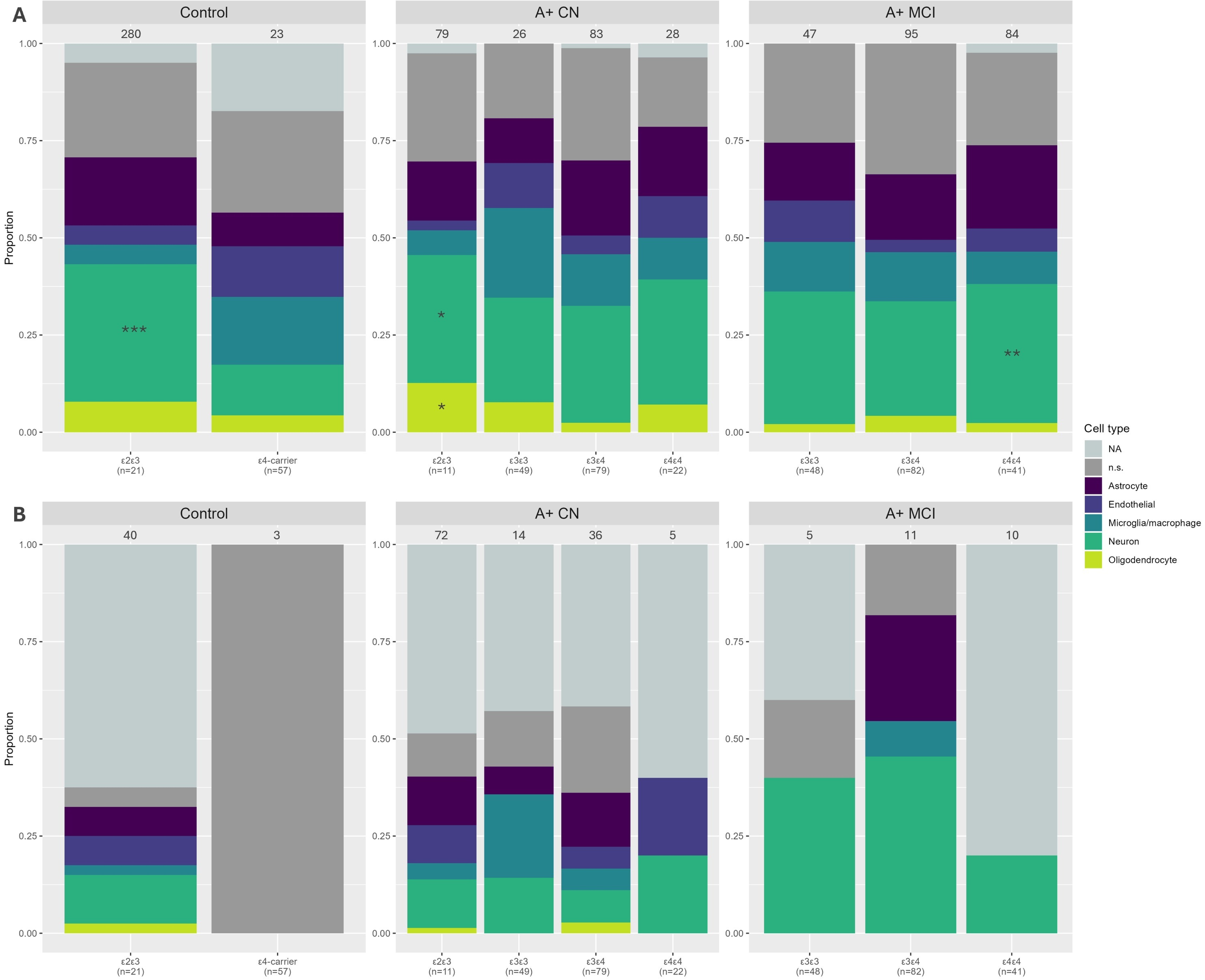

Supplementary Figure 1: Cell-type specificity of proteins associated with each *APOE* genotype in each clinical syndrome, annotated using the RNAseq Barres database (30). The number at the top of each bar represents the total number of proteins with a significant association within that group. The number below each genotype represents the number of individuals in that group. Results are stratified for proteins that exhibit higher (upper row [A]) and lower (lower row [B] levels compared with *APOE* ε3/ε3 controls. *** p-value <0.001, ** p-value <0.01, * p-value <0.05 (Fisher's exact test comparing each number of cell-specific proteins in a specific *APOE* genotype subgroup to the total number of cell-specific proteins measured in our study). *Abbreviations: NA, not available; n.s., non-specific.*
